# High Fat Diet Alters Gut Microbiota and the Expression of Paneth Cell-Antimicrobial Peptides Preceding Changes of Circulating Inflammatory Cytokines

**DOI:** 10.1155/2017/9474896

**Published:** 2017-02-21

**Authors:** Xiulan Guo, Jinchao Li, Renyong Tang, Guodong Zhang, Huawei Zeng, Richard J. Wood, Zhenhua Liu

**Affiliations:** ^1^School of Pharmacy and Biological Engineering, Chengdu University, Sichuan, China; ^2^School of Public Health and Health Sciences, University of Massachusetts, Amherst, MA, USA; ^3^Department of Food Science, University of Massachusetts, Amherst, MA, USA; ^4^Grand Forks Human Nutrition Research Center, Agricultural Research Service, United States Department of Agriculture, Grand Forks, ND, USA; ^5^Jean Mayer USDA Human Nutrition Research Center on Aging, Tufts University, Boston, MA, USA

## Abstract

Obesity is an established risk factor for many diseases including intestinal cancer. One of the responsible mechanisms is the chronic inflammation driven by obesity. However, it remains to be defined whether diet-induced obesity exacerbates the intestinal inflammatory status by cytokines produced in adipose tissue or the high fat diet first alters the gut microbiota and then drives intestinal inflammation. To address this question, we fed C57BL/6 mice with a high fat diet (HF, 60%) and sacrificed them sequentially after 8, 12, and 16 weeks, and then compositions of gut microbiota and expressions of antimicrobial peptides were determined. The compositions of gut microbiota were altered at 8 wk HF feeding, followed with reduced Paneth antimicrobial peptides lysozyme and Reg III*γ* after 12 and 16 wk HF feeding (*p* < 0.05), whereas elevations of circulating inflammatory cytokines IFN*γ* and TNF-*α* were observed until feeding a HF diet for 16 weeks (*p* < 0.05). These results indicated that high fat diet may stimulate intestinal inflammation via altering gut microbiota, and it occurs prior to the potential influence by circulating inflammatory cytokines. These findings emphasized the importance of microbiota, in addition to adipose tissue per se, in driving intestinal inflammation, which may thereafter promote intestinal tumorigenesis.

## 1. Introduction

The prevalence of obesity has increased drastically and reached a pandemic proportion worldwide during recent decades; a further increase is even predicted, ~50% by 2030 [1, 2]. It constitutes an extremely serious threat to the public health since obesity is associated with an array of medical complications, including increased risk of type II diabetes, fatty liver disease, atherosclerosis, and cancers [3]. One of the responsible mechanisms for this relationship is inflammation associated with obesity. It is well accepted that obesity induces a chronic low-grade inflammation [4, 5].

The well-established linkage between chronic inflammation and colorectal cancer (CRC) strongly suggests that inflammation is an important mediator in the obesity-CRC association [6, 7]. A number of studies indicate that inflammatory cytokines are produced by macrophages infiltrated in the adipose tissue in response to enlarged adipocytes [8, 9]. However, in addition to inflammation in adipose tissue per se, obesity is associated with a systematic inflammatory condition [5, 10]. Recently, several studies, including our own, suggest that an inflammatory milieu resides in the colon of obese animals [11, 12]. Although inflammatory cytokines produced in adipose tissue can circulate to the intestinal epithelial layer, a local inflammatory response to the changes of microenvironment, for example, microbiota, in the intestine might also play a critical role in promoting intestinal inflammation and thereby contributing to gastrointestinal diseases. Therefore, a full understanding of origins of intestinal inflammation is needed to dissect the etiology of CRC associated with obesity.

The intestinal innate immune system is one of the most important factors involved in the interactions between gut microbiota and the host. Paneth cells have been shown to be crucial in the prevention of translocation of commensal and pathogenic bacteria [13, 14]. These cells, residing in the crypts of the intestine, produce large amounts of antimicrobial peptides including cryptdin 5, lysozyme, regenerating islet derived 3-gamma (Reg III*γ*), and angiogenin 4, which play a critical role in controlling the microbiota composition of both the small and the large intestine [15, 16]. A shift of microbial composition observed in mice with defects of peptide cryptdins demonstrated a reciprocal relationship exists between gut microbiota and the intestinal physiological state including inflammatory status [17, 18]. Therefore, the altered microbiota, other than adipose tissue per se, might also play a major role in promoting intestinal inflammation in an obese state.

The present study examined the alterations of gut microbial composition, the expressions of Paneth antimicrobial peptides, and inflammation status in a time-course fashion. Our data demonstrated that alterations of gut microbiota were accompanied with the changes of Paneth cell-antimicrobial peptides but preceded the elevations of circulating inflammatory cytokines in high fat diet-induced obese mice, indicating that high fat diet induces alterations of microbiota, which may stimulate intestinal inflammation even before the influence from circulating inflammatory cytokines that were produced in adipose tissue. These findings emphasized the importance of microbiota, in addition to adipose tissue per se, in driving intestinal inflammation, which may thereafter contribute to colorectal tumorigenesis.

## 2. Material and Methods

### 2.1. Animal Study

The animal protocol was approved by the Institutional Animal Care and Use Committee of University of Massachusetts, Amherst. A high fat (HF) diet with 60% kcal from fat (D12492; Research Diets Inc.) was used to induce obesity with 8 C57BL/6 male animals sacrificed at 8, 12, and 16 weeks of feeding. Our previous study [19] has demonstrated a HF diet could successfully induce alterations in both gut microbiota and intestinal inflammation when compared to a LF group after 16 wk feeding, and therefore only one LF control group with 8 wk feeding was designed to determine which alteration, the microbiota or the inflammation, appears first at the early stage. The control group was fed with a low fat (LF) diet with 10% kcal from fat (D12450B; Research Diets Inc.). This 60% kcal HF (with the control 10% kcal LF diet) is widely used for diet-induced obesity [20, 21]. The diet compositions of HF and LF are shown in Table 1.

After 8, 12, and 16 weeks of feeding, 8 male mice were euthanized with CO_2_ at each time point for the HF-fed animals, and the LF control group was terminated after 8 wk feeding. The blood sample was collected via heart puncture after euthanasia, and serum was separated and stored at −80°C for later analysis of inflammatory cytokines. Following with the heart puncture, the abdomen was opened and visceral fat pads were harvested. Samples of the intestinal contents were collected, frozen immediately with dry-ice, and stored at −80°C for further microbial abundance analysis. A segment (~2 cm) of ileum was excised and formalin-fixed for immunohistochemistry, and the intestinal mucosa was collected by gently scraping the rest of the intestine with microscope slides at 0°C and then immediately frozen with dry-ice and stored at −80°C for later analysis of antimicrobial peptides by western blotting or real-time PCR.

### 2.2. Determination of Microbial Profile in the Intestine

To determine the shift of gut microflora, a total of 12 microbial taxa, 4 at the phylum level and 8 at the genus level, were determined by real-time PCR. The 4 phyla (Firmicutes, Bacteroidetes, Actinobacteria, and *Υ*-Proteobacteria) cover more than 90% in the human and mouse gut microbiota [22, 23]. Most of primers used in this study have been previously validated; the remaining primers were designed on the basis of 16S rDNA gene sequences available from the GenBank database and Ribosomal Database Project database (http://rdp.cme.msu.edu/). Primers were synthesized commercially (Invitrogen, Carlsbad, CA), and sequences and relative expressions are listed in Supplementary Data (see* Table S1* in the Supplementary Material available online at https://doi.org/10.1155/2017/9474896).

Bacterial DNA was extracted from intestinal contents using the QIAamp DNA Stool Mini Kit (Qiagen, Valencia, CA) according to the manufacturer's instructions. The concentration and purity of extracted DNA were determined using the NanoDrop-2000 spectrophotometer (ThermoFisher Scientific, Waltham, MA). Real-time PCR assays were performed on the ViiA™ 7 System (Applied Biosystems, Foster City, CA). PCR conditions were 10 min at 95°C, followed by 40 cycles of 95°C for 15 s and 60 s at 60°C.

### 2.3. Real-Time PCR for the Expressions of Antimicrobial Peptides

The expressions of 4 antimicrobial peptides (*lysozyme*,* Reg IIIγ*,* angiogenin 4,* and* cryptdin 5*) and 4 inflammatory cytokines (*TNF-α, IFN-γ, IL-1β,* and* IL-6*) in the intestinal epithelial layer were measured by real-time PCR. Briefly, total RNA was extracted from the small intestine with Trizol (Invitrogen, Carlsbad, CA); the concentrations of total RNA were determined spectrophotometrically (NanoDrop-2000, ThermoFisher Scientific, Waltham, MA), and cDNA was synthesized with SuperScript III (Invitrogen, Carlsbad, CA). Real-time PCR was performed on the ViiA 7 System (Applied Biosystems, Foster City, CA). Primer sequences were listed in Supplementary Data (*Table S2 and Table S3*).

### 2.4. Immunohistochemistry and Western Blotting of Antimicrobial Peptides

Immunohistochemical analysis was performed on the formalin-fixed sections of small intestine. The paraffin-embedding slides were deparaffinized in xylene, followed by rehydration in ethanol. After blocking nonspecific antibody with 5% bovine serum albumin, sections were incubated with the specific 1st antibody of goat polyclonal antibody lysozyme C (W-20) (Santa Cruz Biotechnology, Dallas, TX) followed with the 2nd antibody rabbit anti-goat IgG HRP. Immunoreactivity was detected with horseradish peroxidase-conjugated anti-rabbit EnVision kit (DAKO). All slides were counterstained with hematoxylin.

For western blotting, intestinal epithelial mucosa samples were homogenized in RIPA lysis buffer with protease inhibitor cocktail (Santa Cruz Biotechnology, Dallas, TX). The protein concentration of supernatants was determined using a Pierce™ BCA protein assay kit (ThermoFisher Scientific Inc., Waltham, MA). 40 ug of protein was heated for 5 min in SDS sample buffer, separated by SDS-PAGE on a 12% polyacrylamide gel, and transferred onto polyvinylidene fluoride membrane (Millipore, Bedford, MA). After blocking with 5% nonfat milk, the membrane was incubated with anti-lysozyme antibody (Santa Cruz Biotechnology) or anti-GAPDH antibody (Cell Signaling), and then the HRP-conjugated 2nd antibody (Santa Cruz Biotechnology) was applied. Signals were detected using the ECL Plus Substrate (Amersham Biosciences) on blue X-ray film (ThermoFisher Scientific). Band intensity was analyzed using Image J (ver. 1.49), and the ratio of the density for lysozyme versus GAPDH band was calculated.

### 2.5. Multiplex Inflammatory Cytokine Assays

For the electrochemiluminescent assay of inflammatory cytokines in the blood, 6 cytokines, IFN-*γ*, IL-1*β*, IL-6, IL-2, IL-10, and TNF-*α*, were selected from the U-PLEX panel for mice (Meso Scale Discovery, Rockville, MD), and the assay was performed on the MESO QuickPlex SQ 120 platform following the manufacturer's instructions. All standards and samples were measured in duplicate. The concentrations of inflammatory cytokines were expressed as pg/mL.

### 2.6. Statistical Analysis

Data are expressed as means ± SEM. Data analysis was performed using SAS (version 9.4, SAS Institute, Cary, NC). Comparisons between groups were made using ANOVA and associations were assessed by Pearson's correlation. Heatmaps were created based on transformed *z*-score values using HemI software [24].

For the bacterial data analysis, the abundance of specific bacterial taxa was normalized to total bacteria (ΔCt = Ct_specific_ − Ct_total_), and for the gene expression data analysis, the expression of each gene was normalized to the housekeeping gene *β*-actin (Ct_target  gene_ − Ct_*β*-actin_). Statistical analyses were performed based on ΔCt. The relative abundance of specific bacterial taxa or relative gene expression was reported as 2^−ΔΔCt^, where ΔΔCt = Ct_Experiment_ − Ct_Control_.

## 3. Results

### 3.1. Physiology

The consumption of HF diet for 8 weeks after weaning significantly increased the body weight by 20.3% (*p* < 0.01) compared to the LF group. For those animals with a further 8 wk feeding up to 16 weeks on HF, the body weight incrementally increased 43.0% (*p* < 0.01) when compared to the body weight of animals with only 8 wk HF feeding. Since the gonadal fat pad can be more precisely dissected than other fat pads, we weighed gonadal fat pad and calculated the percentage of it over the total body weight to approximately examine the effect of HF on the fat composition. We observed that both the absolute gonadal fat pad weight and its percentage over total body weight significantly increased when comparing the 8 wk HF group with the 8 wk LF group or the 16 wk HF group with the 8 wk HF group (Table 2).

### 3.2. The Abundances of Gut Microflora

**Figure 1:**
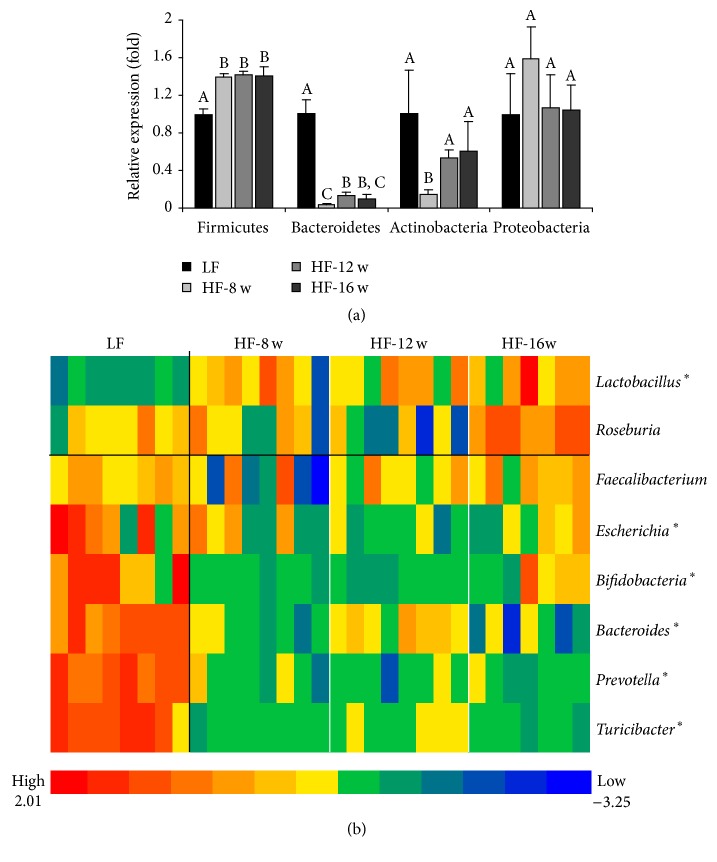
Effect of high fat diet and time course of feeding on the composition of gut microflora. Based on the data of 4 phyla (a) and 8 genera (b) examined, the gut microflora were significantly altered after 8 wk feeding of a high fat diet, and no significantly alterations occurred for further high fat consumption. *∗* means there were significant changes (*p* < 0.05) between HF and LF groups.

Four phyla, which cover 90% of the mouse gut microflora, and 8 genera of gut bacteria were examined in the intestinal content (Figure 1). At the phylum level, HF consumption for 8 weeks increased the Firmicutes and decreased the Bacteroidetes comparing with the LF group (*p* < 0.01), but no further significant change in these phyla was observed after 12 or 16 wk HF feeding (Figure 1(a)). At the genus level, a similar pattern was observed: the gut microflora was altered after 8 wk HF feeding, but no further significant changes were observed in the microbial composition. Mice with HF feeding had higher abundance of genus* Lactobacillus *with reduced abundances of genera* Turicibacter, Prevotella, Bacteroides, *and* Bifidobacteria* (Figure 1(b)).

**Figure 2:**
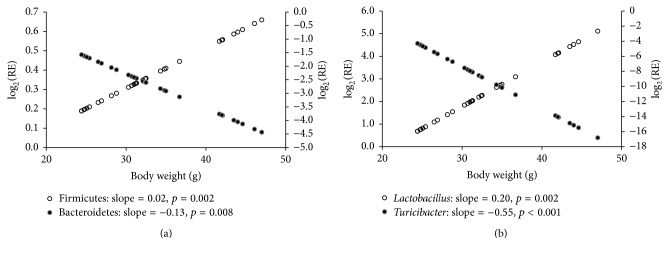
Correlations of body weight with abundances of gut microflora. (a) At the phylum level, the abundance of Firmicutes was positively associated with body weight, whereas the abundance of Bacteroidetes was negatively associated with body weight. (b) At the genus level, the abundance of* Lactobacillus* was positively associated with body weight, whereas the abundance of* Turicibacter* was negatively associated with body weight.

Based on Pearson's correlation analysis of the body weight with abundances of gut microflora, we observed that, at the phylum level, the abundance of Firmicutes (slope = 0.02, *p* = 0.002) was positively associated with body weight, whereas the abundance of Bacteroidetes (slope = −0.13, *p* = 0.008) correlated negatively with body weight (Figure 2(a)). At the genus level, the abundance of* Lactobacillus* (slope = 0.20, *p* = 0.002) was positively associated with body weight, whereas the abundance of* Turicibacter* (slope = −0.55, *p* = 0.001) negatively correlated with body weight (Figure 2(b)).

### 3.3. The Expression of Intestinal Paneth Cell-Antimicrobial Peptides and Inflammatory Cytokines

**Figure 3:**
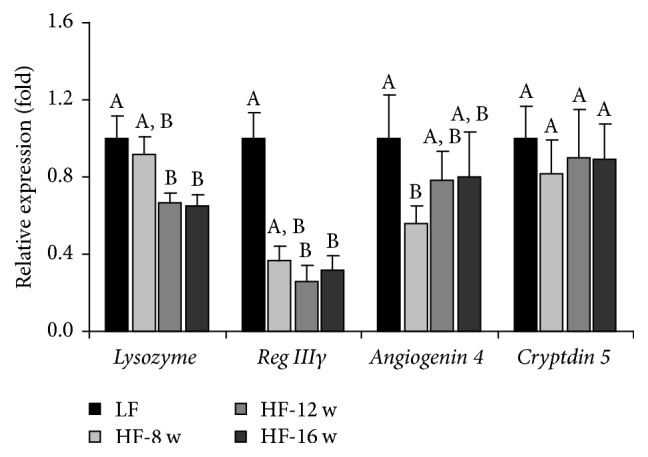
Effect of HF consumption on the relative expression of Paneth cell-antimicrobial peptides. Except for* cryptdin 5*, the mRNA expressions of lysozyme,* Reg IIIγ,* and* angiogenin 4* were significantly reduced at either 8 weeks or 12 and 16 weeks of HF feeding.

**Figure 4:**
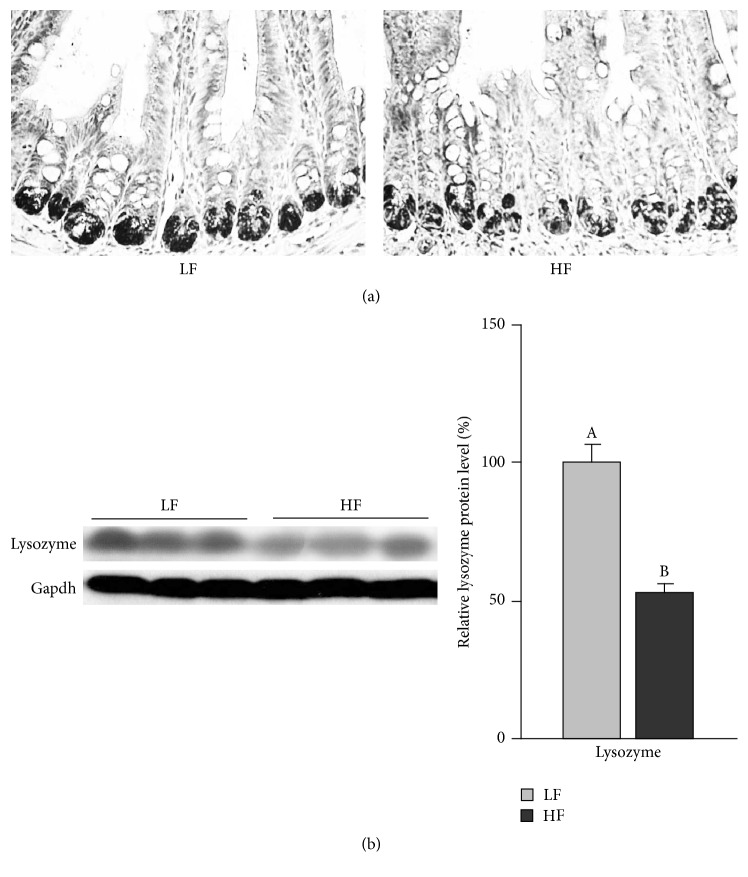
Effect of high fat diet consumption for 16 weeks on the protein levels of lysozyme in the small intestinal epithelial cells. (a) Immunohistochemistry; (b) western blotting.

The mRNA expression of antimicrobial peptides in the small intestinal epithelial cells was examined by real-time PCR. The results indicated that, except for* cryptdin 5*, the mRNA expressions of* lysozyme, Reg IIIγ,* and* angiogenin 4* were significantly reduced at either 8 weeks or 12 and 16 weeks of HF feeding. The data indicated a pattern of reduced expression of these antimicrobial peptides corresponding to the changes of gut microflora as early as 8 weeks of HF feeding (Figure 3). The results for lysozyme protein levels, measured by immunohistochemistry (Figure 4(a)) and western blotting (Figure 4(b)), also indicated a decrease after 16 wk HF feeding.

**Figure 5:**
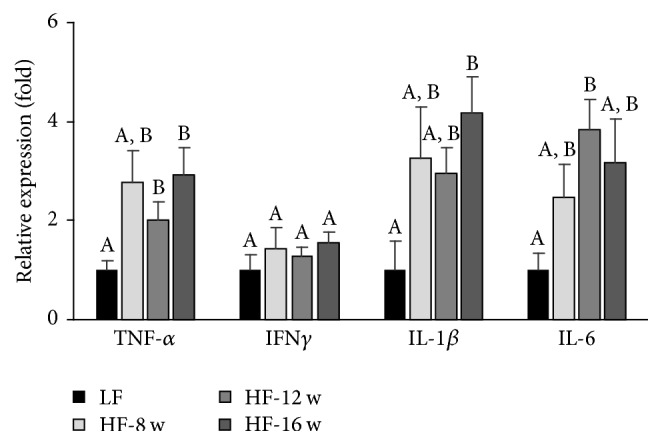
Effect of high fat diet consumption on the relative expression of inflammatory cytokines in the small intestinal epithelial cells. Among the 4 cytokines examined, except for IFN*γ*, the mRNA expressions of TNF-*α*, IL-1*β*, and IL-6 were significantly enhanced at least in one of the three HF groups when compared to the control group fed with a LF diet for 8 weeks.

Among the 4 inflammatory cytokines whose mRNA expressions were examined, except for IFN*γ*, the mRNA expressions of TNF-*α*, IL-1*β*, and IL-6 were significantly enhanced at least in one of the three HF groups (HF-8, HF-12, and HF-16) when compared to the control group fed with a LF diet for 8 wks. A pattern of increased expressions of TNF-*α*, IL-1*β*, and IL-6 in the HF groups was clearly shown in Figure 5. This data indicated a local production of inflammatory cytokines in the intestinal epithelial layer.

### 3.4. The Profile of Circulating Inflammatory Cytokines

**Figure 6:**
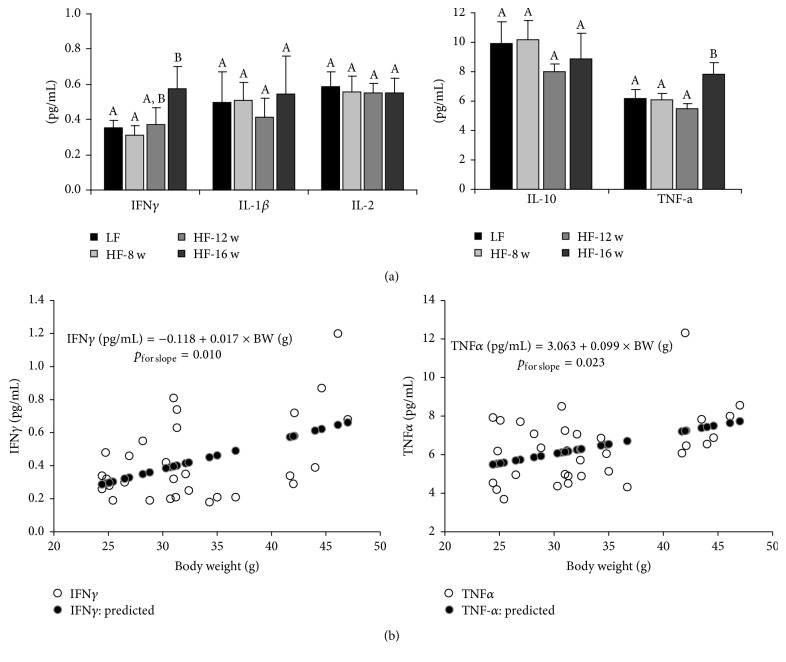
Effect of high fat diet consumption on circulating inflammatory cytokine profile. (a) Serum inflammatory cytokine levels across the groups. (b) Pearson's correlations of IFN*γ* and TNF-*α* with body weight.

Six serum inflammatory cytokines, IFN-*γ*, IL-1*β*, IL-6, IL-2, IL-10, and TNF-*α*, were profiled using the multiplex assay (Meso Scale Discovery, Rockville, MD). Except for an unexpected nondetection of IL-6, among the other 5 cytokines, we did not observe significant changes for the 8 wk and 12 wk HF groups when comparing to the 8 wk LF group, whereas the levels for serum IFN*γ* and TNF-*α* in the 16 wk HF animals were significantly increased when compared to the LF group (Figure 6(a)), indicating the changes of serum inflammatory cytokines were later than the changes of gut microflora, which occurred at 8 weeks of HF feeding as described above. When Pearson's analysis was performed, significantly positive correlations were observed for both IFN*γ* (*p* = 0.010) and TNF-*α* (*p* = 0.023) with body weight (Figure 6(b)). Every 10-gram increase of body weight corresponds to an increase of 0.17 pg/mL for IFN*γ* and 0.99 pg/mL for TNF-*α*, which can be translated into 47% increase for IFN*γ* and 16% increase for TNF-*α*, if the levels in the LF group were set as the baseline.

## 4. Discussion

An inflammatory milieu in the intestine of obese animals has been recently demonstrated [11, 12]. The increased inflammatory cytokines in the intestinal epithelia may result from the cytokines produced in adipose tissue or are induced by a local inflammatory response to the changes of intestinal microenvironment in an obese state. The present study demonstrated that HF altered gut microbiota corresponding with changes of Paneth cell-antimicrobial peptides, but preceding the elevations of circulating inflammatory cytokines. These results indicated that HF may stimulate intestinal inflammation via altering gut microbiota, and this occurs before the potential influence by inflammatory cytokines produced in adipose tissue elsewhere in the body. The findings emphasized the importance of microbiota, in addition to adipose tissue per se, in driving intestinal inflammation in an obese state.

A close relationship between the intestinal microbiota and obesity has been demonstrated in both human and animals, with a large shift in microbiota favoring the presence of Firmicutes and suppressing Bacteroidetes in obesity [22, 23]. Studies have demonstrated that a high fat diet [25] and a high fat/high-sucrose diet [26] promoted a decrease in Bacteroidetes and an increase in Firmicutes, a shift observed in obese individuals [23]. However, it remains to be defined whether the alteration in gut microbial composition results directly from the HF per se or from the altered intestinal microenvironment in an obese state, for example, the promoted inflammatory status by the circulating cytokines. The present study demonstrated that the alterations of the gut microbial composition occurred prior to the elevation of circulated inflammatory cytokines, indicating HF directly influences gut microbial composition. These findings reemphasize the importance of high fat diet by itself in altering gut microbial composition, not the obese state [27]. Indeed, some studies demonstrated that diet, rather than other factors, such as host genotype, plays a dominant role in shaping gut microbial communities [28, 29].

High fat diet creates a shift in the composition of the gut microbiota and generally corresponds with diminished expressions of Paneth antimicrobial peptides [30–32]. In this study, changes in the gut microbial profile occurred after 8 weeks of HF feeding and reduced expressions of lysozyme and Reg III-*γ* were observed after 12 weeks of HF feeding, indicating that the alteration of Paneth antimicrobial peptides was secondary to the shift of microbiota, whereas alterations of circulating inflammatory cytokines (IFN*γ* and TNF-*α*) were only observed after 16 weeks of HF feeding in this study. These findings indicated that HF directly altered the profile of gut microbiota and the expressions of Paneth antimicrobial peptides that may stimulate intestinal inflammation before the potential influence by circulating inflammatory cytokines. These results demonstrated the role of HF-altered gut microbiota in stimulating intestinal inflammation, but it is noteworthy that the systematic inflammation associated with obesity should originate from both adipose tissue [8, 9] and intestinal mucosa [11] as shown by the increased gene expression of inflammatory cytokines in those tissues. Our gene expression data for inflammatory cytokines demonstrated an increased local production in the intestinal epithelial layer associated with HF diet feeding.

Similar to the previous studies [23, 25, 27], our study found that HF feeding reduced significantly the Bacteroidetes and increased the Firmicutes comparing to the LF control at 8 weeks. The gut microbiota was altered after 8 weeks of HF feeding and stabilized afterwards. However, it is highly noteworthy that although the phylum Firmicutes was significantly increased along with HF feeding, the abundance of* Turicibacter, *a genus within the Firmicutes phylum, was dramatically decreased (Figures 1 and 2). Consistent with the observation in this study, data from another study in our lab [33] and others [34] also showed that the abundance of* Turicibacter *was decreased markedly in mice fed a high fat diet. A couple of studies showed a depletion of* Turicibacter* in animal models of inflammatory bowel disease [35, 36], and Zhong et al. [37] found that the content of butyric acid, an important short chain fatty acid with antimicrobial property [38], was positively correlated with* Turicibacter*. These findings raise an important question whether this genus possesses the capability to produce butyric acid and has probiotic properties.

## 5. Conclusions

In summary, the data from our time-course HF feeding animal study clearly demonstrated that HF altered gut microbial communities, corresponding with the changes of Paneth cell-antimicrobial peptides but prior to the elevations of circulating inflammatory cytokines, indicating that high fat diet induces alterations of microbiota, which may stimulate intestinal inflammation even before the influence from circulating inflammatory cytokines that were produced in adipose tissue elsewhere in the body. These findings suggested that gut microbiota, rather than adipose tissue per se, may play a primary role in driving intestinal inflammation in diet-induced obesity.

## Supplementary Material

Table S1: Primer sequences for microbes; Table S2: Primer sequences for anti-microbial peptides; Table S3: Primer sequences for inflammatory cytokines.

## Figures and Tables

**Table 1:** The compositions of high fat and low fat diets used in this study.

	LF	HF
*Ingredient (g/kg)*		
Casein	189.6	258.5
L-Cystine	2.8	3.9
Corn starch	298.6	0
Maltodextrin	33.2	161.5
Sucrose	331.8	88.9
Cellulose	47.4	64.6
Soybean oil	23.7	32.3
Lard	19.0	316.6
Mineral mix S10026	9.5	12.9
Dicalcium phosphate	12.3	16.8
Calcium carbonate	5.2	7.1
Potassium citrate	15.6	21.3
Vitamin mix V10001	9.5	12.9
Choline bitartrate	1.9	2.6
Total	1000	1000

*Energy (% kcal)*		
Protein	20	20
Carbohydrate	70	20
Fat	10	60
Total	100	100

Diets: LF, low fat diet. HF, high fat diet. Research Diets Products # D12450B and D12492, respectively.

**Table 2:** Effect of diet and time on body weight and fat pad.

Groups	Body weight (g)	Gonadal fat (g)	Gonadal fat (%)
LF	25.50 ± 0.48^a^	0.61 ± 0.08^a^	2.39 ± 0.30^a^
HF-8 w	30.69 ± 0.73^b^	1.15 ± 0.14^b^	3.70 ± 0.39^b^
HF-12 w	33.05 ± 0.85^b^	1.74 ± 0.24^bc^	5.20 ± 0.66^c^
HF-16 w	43.88 ± 0.69^c^	2.29 ± 0.19^c^	5.21 ± 0.43^c^

Data are expressed as mean ± SEM. In each column, the values with different letters indicate significant difference among groups (*p* < 0.05).
