# Supplementary material for: High Fat Diet Alters Gut Microbiota and the Expression of Paneth Cell-Antimicrobial Peptides Preceding Changes of Circulating Inflammatory Cytokines
Source: Mediators Inflamm. 2017 Feb 21;2017:9474896. doi: 10.1155/2017/9474896 (PMC5339499; doi:10.1155/2017/9474896)
Supplement: Supplementary file 1 — Table S1: Primer sequences for microbes; Table S2: Primer sequences for anti-microbial peptides; Table S3: Primer sequences for inflammatory cytokines. [file 9474896.f1.docx]

**Supplementary Data_Table S1:** Primer sequences and relative abundances of microbes resident in small intestine of mice when comparing the LF group with the combined high fat groups.

| **Name** | **Forward Primer** | **Reverse Primer** | | **Low Fat** | **High Fat^#^** |
| --- | --- | --- | --- | --- | --- |
| *Bacteroidetes* | GGARCATGTGGTTTAATTCGATGAT | AGCTGACGACAACCATGCAG | | 1.000±0.015 | 0.119±0.022** |
| *Firmicutes* | GGAGYATGTGGTTTAATTCGAAGCA | AGCTGACGACAACCATGCAC | | 1.000±0.054 | 1.419±0.033** |
| *Proteobacteria* | TCGTCAGCTCGTGTYGTGA | CGTAAGGGCCATGATG | | 1.000±0.436 | 1.506±0.190 |
| *Actinobacteria* | TACGGCCGCAAGGCTA | TCRTCCCCACCTTCCTCCG | | 1.000±0.939 | 0.508±0.116 |
| *Lactobacillus* | GCAGCAGTAGGGAATCTTCCA | GCATTYCACCGCTACACATG | | 1.000±0.137 | 13.816±3.630** |
| *Roseburia* | GCGGTRCGGCAAGTCTGA | CCTCCGACACTCTAGTMCGAC | | 1.000±0.205 | 1.512±0.254 |
| *Faecalibacterium* | GGAGGAAGAAGGTCTTCGG | AATTCCGCCTACCTCTGCACT | | 1.000±0.091 | 0.092±0.130 |
| *Prevotella* | AGCCAAGTAGCGTGCAGGAT | CTGCTGGCACGGAATTAGC | | 1.000±0.115 | 0.051±0.006** |
| *Bacteroides* | CCTWCGATGGATAGGGGTT | CACGCTACTTGGCTGGTTCAG | | 1.000±0.193 | 0.110±0.019** |
| *Bifidobacterium* | CGGGTGAGTAATGCGTGACC | TGATAGGACGCGACCCCA | | 1.000±10.439 | 0.171±0.168** |
| *Escherichia* | CATGCCGCGTGTATGAAGAA | CGGGTAACGTCAATGAGCAAA | | 1.000±0.747 | 0.166±0.053** |
| *Turicibacter* | CAGACGGGGACAACGATTGGA | TACGCATCGTCGCCTTGGTA | | 1.000±0.893 | 0.0003±0.0001** |
| ***16S rDNA*** | ACTCCTACGGGAGGCAGCAG | | ATTACCGCGGCTGCTGG | N/A | N/A |

**^#^**Since there were no significant pattern changes among the groups with 8-, 12- and 16-weeks of high fat diet feeding, and therefore these groups are combined.

* Indicates a difference set at *p* < 0.05, ** Indicates a difference set at *p* < 0.01 when comparing High fat group with Low fat control.

**Supplementary Data_Table S2:** Primer sequences used for the quantification of the expressions of anti-microbial peptides in small intestinal epithelial cells byreal-time quantitative PCR.

| **Genes** | **Forward Primer** | **Reverse Primer** | **Reference Sequence No.** |
| --- | --- | --- | --- |
| Lysozyme | AGATCCCCAAGGCATTCGA | TCCTACAGTGAGAAAGAGACMGAAT | AK150998.1 |
| Cryptdin 5 | TGGGCTCCTGCTCAACAATT | TGCTCCTCAGTATTAGTCTCTTCATCTG | NM_007851.2 |
| Angiogenin 4 | TGTCCTTTGTTGTTGGTCTTCGT | GTGCTGACGTAGGAATTTTTCGT | BC042938.1 |
| RegIIIγ | CCTTCCTGTCCTCCATGATCA | CCACTCCCATCCACCTCTGT | NM_011260.1 |
| β-Actin | GGCTGTATTCCCCTCCATCG | CCAGTTGGTAACAATGCCATGT | NM_007393.5 |

**Supplementary Data_Table S3:** Primer sequences used for the quantification of the expressions of inflammatory cytokines in small intestinal epithelial cells by real-time quantitative PCR.

| **Genes** | **Forward Primer** | **Reverse Primer** | **Reference Sequence No.** |
| --- | --- | --- | --- |
| TNF-α | AGGGTCTGGGCCATAGAACT | CCACCACGCTCTTCTGTCTAC | NM_013693.3 |
| IFN-γ | TGAGCTCATTGAATGCTTGG | ACAGCAAGGCGAAAAAGGAT | NM_008337.4 |
| IL-1β | GGTCAAAGGTTTGGAAGCAG | TGTGAAATGCCACCTTTTGA | NM_008361.4 |
| IL-6 | ACCAGAGGAAATTTTCAATAGGC | TGATGCACTTGCAGAAAACA | NM_031168.2 |
| β-Actin | GGCTGTATTCCCCTCCATCG | CCAGTTGGTAACAATGCCATGT | NM_007393.5 |
